# Math Anxiety and Metacognition: How Numerical Content Shapes Confidence

**DOI:** 10.3390/jintelligence14100237

**Published:** 2026-10-02

**Authors:** Yael Sidi, Kinga Morsanyi, Rakefet Ackerman

**Affiliations:** 1 Department of Education and Psychology, The Open University of Israel, Ra’anana 4353701, Israel; 2 Department of Mathematics Education, Loughborough University, Loughborough LE11 3TU, UK; k.e.morsanyi@lboro.ac.uk; 3 Faculty of Data and Decision Sciences, Technion–Israel Institute of Technology, Haifa 3200003, Israel; ackerman@technion.ac.il

**Keywords:** calibration, cognitive reflection, confidence, math anxiety, metacognitive monitoring, reasoning

## Abstract

Math anxiety is robustly linked to lower success rates in mathematical tasks, yet its relation to metacognitive aspects, such as confidence and overconfidence, remains less well understood. Moreover, prior research often used tasks in which numerical content and mathematical reasoning were intertwined, making it difficult to determine whether math anxiety is triggered by computation itself or by numerical framing more broadly. To address these gaps, two experiments examined associations between math anxiety, task success rates, confidence, and overconfidence. In both experiments, participants completed a novel Single-Answer Sudoku Task (SAST) and the Cognitive Reflection Test (CRT), together with anxiety-related self-report questionnaires. In Experiment 1 (*N* = 209), the SAST contained numbers, similar to the traditional Sudoku. Math anxiety predicted lower success rates and confidence, and greater overconfidence, in both the numerical SAST and the CRT, beyond relevant individual-difference variables. Experiment 2 (*N* = 200) used a structurally identical SAST, in which letters replaced numbers. While the CRT results replicated Experiment 1, math anxiety no longer predicted confidence or overconfidence in the letter SAST. The findings suggest that math anxiety relates to impaired metacognitive monitoring when numerical content is present, even without computational demands, but not in equivalent non-numerical reasoning tasks.

## 1. Introduction

Research in cognitive psychology and education has long demonstrated that variability in negative affective responses to mathematics, termed math anxiety, is associated not only with achievement (Dowker et al., 2016; Luttenberger et al., 2018; Zhang et al., 2019), but also with patterns of persistence (e.g., Jenifer et al., 2022), and avoidance of math-related tasks, activities, and careers (e.g., Choe et al., 2019; Dowker et al., 2016; Lau et al., 2024; Luttenberger et al., 2018). Understanding the relationship between math-related self-perceptions and thinking can be enriched by delving into process indicators and boundaries of these effects.

One possibility is that math anxiety is only triggered when mathematical symbols are combined with requirements for computations or mathematical reasoning (e.g., arithmetic, algebra). A second possibility is that math anxiety reflects sensitivity to numerical features even when people are faced with non-mathematical reasoning tasks that include numerical symbols (e.g., Sudoku). The latter option would be in line with the original conceptualization of math anxiety as “number anxiety” (Dreger & Aiken, 1957). Finally, it is possible that math anxiety relates to performance on reasoning tasks that resemble numerical tasks (for example, due to structural similarities), even when they do not have numerical content.

The present study aimed at distinguishing between these three possibilities. To do this, we introduce hereby an adapted version of the classic numerical Sudoku task and a structurally equivalent, letter-based task. We employed the metacognitive approach (Ackerman & Thompson, 2017; Nelson & Narens, 1990) to delve into process indicators using item-by-item confidence, beyond task success. By comparing numerical and non-numerical versions of the same task, we examined the specificity of math anxiety’s relationship with process indicators and outcomes.

### 1.1. Math Anxiety

Math anxiety is a feeling of tension, apprehension, and fear that interferes with mathematical performance in learning and everyday contexts (Ashcraft & Ridley, 2005). Large-scale international assessments suggest that math anxiety is widespread and approximately normally distributed in the general population (Hart & Ganley, 2019), with over 90% of adults reporting at least some level of math anxiety, whereas approximately 10% to 20% fall within a high-anxiety range, depending on measurement and cutoff criteria (Luttenberger et al., 2018; Megreya et al., 2024). Math anxiety emerges early in childhood (e.g., Primi et al., 2020, Ramirez et al., 2013; Retanal & Maloney, 2026) and tends to increase across the school years, particularly as mathematical tasks become more complex, evaluative and socially comparative (Dowker et al., 2016). Longitudinal evidence suggests that early negative experiences with mathematics, such as repeated failure, negative feedback, or classroom environments emphasizing speed and accuracy, contribute to the development and stabilization of math anxiety (Carey et al., 2016; Wang et al., 2020). Math anxiety also interferes with mathematics learning, starting from the first school years, potentially leading to cumulative gaps in knowledge (Tomasetto et al., 2021). Socialization processes, including parental attitudes toward mathematics and their active involvement in helping with math homework, have also been shown to play a role, particularly in shaping children’s beliefs about their own mathematical competence (Maloney et al., 2015, but see Tomasetto et al., 2025). Once established, math anxiety often persists into adolescence and adulthood, influencing educational choices and occupational trajectories (Hembree, 1990; Ramirez et al., 2018).

Although math anxiety can be experienced as a situational response, it is usually conceptualized and measured as a stable individual characteristic with trait-like properties, showing consistency across time and tasks (Guo & Liao, 2025; Orbach et al., 2019). Importantly, it is regarded as a domain-specific form of anxiety that is distinct from, though correlated with, test anxiety, and general anxiety (Ashcraft & Ridley, 2005; Dowker et al., 2016; Hembree, 1990; Kazelskis et al., 2000). For example, empirical evidence demonstrates that math anxiety uniquely predicts math performance even when controlling for test anxiety and general anxiety (e.g., Chan & Tang, 2020; Hart & Ganley, 2019). Math anxiety is also correlated with subjective numeracy (Rolison et al., 2020). While math anxiety refers to feelings of tension and fear in math contexts, subjective numeracy reflects self-perceptions, liking, and preference for numeric information (Fagerlin et al., 2007), with the two reflecting distinct constructs (Bjälkebring, 2019).

Math anxiety has been robustly and consistently related to poorer math performance (for meta-analyses, see, e.g., Barroso et al., 2021; Namkung et al., 2019), and much research has focused on understanding the mechanisms that underlie this impact. One explanation has centered on *working memory capacity*. For example, the *processing efficiency theory* (Eysenck & Calvo, 1992; Luttenberger et al., 2018) suggests that math anxiety consumes working-memory resources by diverting attention from task-relevant processing toward threat-related information. This diversion harms performance, especially in complex problems, and under conditions that already overload working memory, such as time pressure (Ashcraft, 1995; Ashcraft & Kirk, 2001; Chan & Tang, 2020). Another explanation, the *Deficit Theory* (Carey et al., 2016) proposes that some people have an innate weakness in basic numerical processing (e.g., comparing symbolic magnitudes), which makes math reasoning objectively harder, leading to elevated math anxiety (Maloney, 2016; O’Connor et al., 2023). Notably, research shows support for both explanations, suggesting that a single account is insufficient to fully explain the relations between math anxiety and lower math performance (Skagerlund et al., 2019). Finally, *math avoidance* has also been suggested as a key factor, indicating that repeated avoidance of math creates a cycle in which math skills decrease and anxiety increases (Lau et al., 2024).

While acknowledging the role of all these mechanisms, we focus our investigation on yet another angle, taking a metacognitive approach. Specifically, the *Meta-Reasoning framework* provides conceptualizations and process indicators, allowing us to examine individuals’ metacognitive processes when they face reasoning challenges in general (Ackerman & Thompson, 2017). Mathematical problem solving is a specific case of reasoning that can be examined under this framework (cf., Morsanyi et al., 2019).

### 1.2. Meta-Reasoning and Math Anxiety

According to the classic framework proposed by Nelson and Narens (1990), metacognition comprises two core components: *monitoring*, the process by which individuals judge the quality of their ongoing cognition (e.g., “*How confident am I in my answer?*”), and *control*, the regulatory decisions enacted on the basis of those judgments (e.g., allocating additional thinking time, seeking help). Empirical measures for monitoring rely on subjective self-judgments. For example, participants are asked to judge their ability to solve a problem or judge their confidence in their response. Empirical research has repeatedly demonstrated both the potential for accurate self-judgments (e.g., Dunlosky & Bjork, 2013) and the prevalence of systematic biases in these judgments, reflected in a calibration measure that describes the gap between objective performance and subjective self-judgments (see Ackerman, 2019; Bjork et al., 2013, for reviews). Biased monitoring leads to inefficient control processes, for example, seeking help in case of doubt. Thus, understanding the determinants of monitoring accuracy is central for shedding light on self-regulated learning and performance.

The *cue-utilization theory* provides a theoretical account of how monitoring judgments are formed (Ackerman, 2019; Koriat, 1997), suggesting that rather than directly accessing one’s actual knowledge, individuals infer their own cognition from heuristic cues. Each cue carries a particular cue validity, which is the degree to which it correlates with actual performance, and individuals weigh these cues to assess their knowledge and understanding (Ackerman, 2023). Notably, while these cues can be valid, showing associations with performance (Benjamin et al., 1998; Hertwig et al., 2008), they can also bias the monitoring process (see Finn & Tauber, 2015 for a review). Thus, cue validity carries high significance for self-regulated learning. Importantly, cue-utilization theory emphasizes that monitoring judgments are informed not only by task-based cues, such as perceived difficulty, but also by individuals’ beliefs about their own abilities and traits (e.g., “I am terrible at math”, Ackerman, 2019). Math anxiety can be understood within this framework, as reflecting self-beliefs and affective dispositions related to numerical tasks (Morsanyi et al., 2019). Specifically, assuming that math-anxious people have low self-belief in their abilities to solve math problems, it is expected that they lower their judgments regarding the solvability of math problems, and when assessing their progress towards reaching a solution.

The *Regulated Attention in Mathematical Problem-Solving* framework (RAMPS; Scheibe et al., 2023) provides a process-level model of how math anxiety, working memory, and metacognitive processes interact during mathematical problem solving. Within this model, individuals rely on momentary cognitive and affective cues (e.g., perceived difficulty, effort) to generate monitoring judgments, which, in turn, guide control decisions such as persistence, strategy switching, or disengagement. Math anxiety is posited to distort the interpretation of these cues, biasing monitoring toward lower confidence and shifting control toward avoidance or early disengagement. Over time, this cycle is theorized to reduce exposure to successful problem-solving, thereby reinforcing negative self-beliefs and sustaining math anxiety (Scheibe et al., 2023).

Empirical evidence from health and risk-related numerical problems is consistent with this interpretation, showing that math anxiety relates to the monitoring process and is overall associated with lower confidence (e.g., Desender & Sasanguie, 2022; Rolison et al., 2016, 2020; Scheibe et al., 2022). For example, Scheibe et al. (2022) investigated how educational training affects problem-solving accuracy and metacognitive monitoring regarding health-related math reasoning. Participants were randomly assigned to one of two experimental conditions: an educational intervention in which they received a worked example for calculating and comparing case-fatality rates, and a control condition in which they were provided with the raw health statistics, but received no intervention. Higher math anxiety was consistently associated with lower confidence, when controlling for accuracy, trait anxiety, gender, and numeracy skills. Although the worked-example intervention increased overall confidence, math anxiety continued to explain unique variance in item-level judgments across experimental conditions. Similarly, Desender and Sasanguie (2022) found a negative correlation between math anxiety and confidence, both when measured before solving math problems, and after solving them. Rolison et al. (2016) conducted two studies in which participants solved math-based health problems. In both studies, math anxiety continued to predict confidence when controlling objective numeracy.

Nevertheless, the association between math anxiety and metacognitive measures is not necessarily straightforward. For instance, some studies examined the association between math anxiety and the resolution measure (e.g., within-participant correlation between success and confidence in each task item), reflecting confidence discrimination between correctly and incorrectly solved problems. Desender and Sasanguie (2022) found that math anxiety predicted better discrimination. Erickson and Heit (2015) asked both high school students (Study 1) and undergraduates (Study 2) to solve shortened SAT tests in biology, literature, and math. As in many other cases (e.g., West & Stanovich, 1997), they found consistent overconfidence in predicting performance across all domains. Importantly, overconfidence was higher in math compared to the other domains. Bellon et al. (2021) investigated both concurrent and longitudinal relations between mathematics anxiety, metacognitive monitoring (i.e., calibration), and arithmetic achievement in 7- to 8-year-old children over a 1-year period. Bellon et al. (2021) found both concurrent and longitudinal relations between math anxiety and metacognitive monitoring, with early mathematics anxiety predicting later metacognitive monitoring, but not vice versa. The above studies suggest that math anxiety may affect metacognitive judgments, and especially metacognitive monitoring, in numerical tasks (see Morsanyi et al., 2019; Scheibe et al., 2023, for reviews), although it is currently unclear if math anxiety relates to increased or decreased confidence.

A key limitation of this literature is that numerical content and mathematical reasoning are often intertwined. Most studies examining math anxiety and metacognitive monitoring use tasks that require calculations, proportional reasoning, or other mathematical procedures. Consequently, it remains difficult to determine whether math anxiety affects monitoring because the task requires mathematical reasoning, or because the task contains numerical cues that are perceived as math-related. This distinction is important because emerging evidence suggests that numerical symbols may themselves elicit affective and attentional responses among individuals with high math anxiety, even in the absence of explicit computation. Neuropsychological evidence provides converging, though largely indirect, support for the possibility that math anxiety operates at earlier and more general stages of numeric processing. Specifically, passive exposure to digits has been shown to trigger threat-related neural activity, consistent with an attentional bias toward number symbols (Batashvili et al., 2020). Similarly, studies demonstrate that for adults with high math anxiety, numerical errors evoke stronger error-monitoring and affective responses than comparable non-numerical errors, as well as domain-specific impairments in inhibitory control during simple number tasks (Núñez-Peña & Campos-Rodríguez, 2024; Suárez-Pellicioni et al., 2013). Complementary evidence from working memory research indicates that maintaining and retrieving numeric information disproportionately engages affective and control-related neural networks in individuals with high math anxiety, even when no arithmetic operations are required (e.g., Pelegrina et al., 2020; van Dijck et al., 2022). These findings suggest that even in the absence of explicit computational demands, numeric stimuli tend to elicit heightened affective and attentional responses in adults with high math anxiety.

### 1.3. The Methodological Challenge: Isolating Core Reasoning Processes from Numerical Content Effects

The current study aimed at clarifying the relations between math anxiety and cognitive and metacognitive processes, and the boundary conditions of these relations. Notably, in educational and everyday situations, numbers may appear in different contexts, often combining requirements of mathematical (e.g., knowledge of math-related procedures, the ability to understand graphical representations) and non-mathematical reasoning (e.g., everyday knowledge, text comprehension; e.g., Daroczy et al., 2015; Reinhold et al., 2020; Tout et al., 2021). For instance, algebraic word-problems not only require algebraic procedures, but also the comprehension of temporal, spatial, or relational language, in order to construct an appropriate representation of the problem before creating equations (e.g., Soneira et al., 2023).

Consistently, recent work shows that the relation between math anxiety and performance depends on specific numerical task features. Mielicki et al. (2023) found that this relation varied as a function of number type, symbolic versus non-symbolic format, and component size. These findings suggest that math anxiety is not uniformly related to all numerical tasks, but may be especially sensitive to the features that make a task appear more mathematical. The present study builds on this logic by asking whether numerical symbols themselves, separated from computational demands, are sufficient to elicit math-anxiety-related differences in performance and metacognitive monitoring.

To examine whether math anxiety is tied specifically to mathematical reasoning, or whether it is also triggered by numerical content itself, a reasoning task was required in which the presence of numerical content can be manipulated without changing the structure of the task. The Sudoku task provides an opportunity to examine the effects of the mere presence of numbers on thinking processes, as the puzzles include numbers without actually requiring mathematical computations or background knowledge, beyond mere familiarity with numbers from 1 to 9 (Lee et al., 2008). Building on this logic, we developed a novel variation of the classic Sudoku task, the Single-Answer Sudoku Task (SAST; Figure 1).

### 1.4. The Single-Answer Sudoku Task (SAST)

The classic Sudoku task involves solving a logic-based puzzle consisting of a 9 × 9 matrix, which is further divided into nine 3 × 3 sub-matrices. The goal is to fill the entire matrix with digits (1 to 9) in a way that each digit appears exactly once in each row, column, and 3 × 3 sub-matrix (Lee et al., 2008).

For the present study, we developed the Single-Answer Sudoku Task (SAST)—a novel variation of the classic Sudoku task (see details about task development in Appendix A). Participants’ task was to identify which digit fitted a target cell. Responding was done by entering a single digit as the answer, which makes scoring straightforward. For a metacognitive study to elicit varying levels of confidence, we needed a task in which responses are often provided with uncertainty. The 9 × 9 matrix was presented with most cells already populated, while a few were empty (see Figure 1). Importantly, for triggering a sense of uncertainty, the SAST does not require completing the entire matrix. Furthermore, by isolating a single target cell (the red cell) while leaving some areas incomplete, we transformed a lengthy, recreational puzzle into a highly controlled, standardized cognitive task. The opportunity to collect data from multiple task items from each participant increases statistical power and allows for within-participant correlations as part of the metacognitive analysis (e.g., Ackerman et al., 2016).

Prior work in meta-reasoning has shown that problem solving is guided by early, heuristic-based impressions about difficulty and solvability, which influence effort regulation (Lauterman & Ackerman, 2019, 2024). We manipulated difficulty and perceived difficulty of the puzzles by manipulating the total number of empty cells (Figure 1), and, among them, the number of empty cells that needed to be solved to determine the target number. Uniquely for the math anxiety literature, we increased the challenge even more by including three *unsolvable matrices*. These matrices were generated by violating the Sudoku rules (e.g., a missing digit in one column already appearing in the corresponding row). Participants had to respond by entering “0” if they thought a puzzle was unsolvable.

The task included 18 SAST puzzles, including three very easy puzzles (>90% success), 12 puzzles with success levels below 80%, and three unsolvable puzzles, all randomly ordered. In addition to the numerical version, used in Experiment 1, for Experiment 2, we developed an identical non-numerical version, the letter SAST, in which we replaced each number with the corresponding letter from the English alphabet, ensuring that the task structure and reasoning demands remained equivalent, while removing numerical content. (1 → A, 2 → B, 3 → C, …, 9 → I, see Figure 1). The full set of puzzles and their level of difficulty and solvability can be found in: https://osf.io/2tvxw/ (accessed on 9 September 2026).

To examine whether explicit knowledge about the presence of unsolvable puzzles would shape participants’ interpretation of difficulty and regulation of effort, a notification manipulation was introduced. Participants in the notification condition were informed that some puzzles were unsolvable, whereas participants in the no-notification condition were not given this information, preserving ambiguity about whether difficulty reflected task demands or personal problem-solving competence.

### 1.5. Overview of the Present Study

Our aim was to investigate the impact of the mere presence of numerical content on success rates and metacognitive monitoring. To this end, we compared performance and metacognitive judgments across three task contexts: the numerical SAST, the letter SAST, and the Cognitive Reflection Test (CRT). The SAST and CRT tasks differ in their mathematical demands. The CRT consists of a set of verbal math problems, designed to elicit a conflict between a specific, intuitively compelling, but incorrect response, and a correct response. Importantly, success rates on the CRT have been found to be robustly related to mathematics anxiety, beyond the effects of other forms of anxiety, in diverse populations (e.g., Morsanyi et al., 2014; Mahak et al., 2025). The 6-item variation of the Cognitive Reflection Test (CRT, Frederick, 2005; based on Shenhav et al., 2012 and Primi et al., 2016; see Appendix B for the full list of problems) was included in both experiments, and was always presented after the SAST. This design allowed us to examine the associations between math anxiety and a set of cognitive and metacognitive measures, in the numerical SAST (Experiment 1; numerical content but no numerical reasoning), a structurally equivalent letter SAST (Experiment 2; no numerical content, no numerical reasoning), and the CRT (in both experiments; numerical content and numerical reasoning).

The dependent measures in both the SAST and CRT were success rates, confidence, and overconfidence. Individual differences measures included math anxiety, generalized anxiety, subjective numeracy, and gender. Generalized anxiety was included as a control measure to determine the specific relevance of math anxiety beyond more generalized forms of anxiety. The effect of subjective numeracy, which measures mathematics-related self-concept and self-efficacy, was also taken into account, given its relevance to confidence judgments. The effects of gender were controlled for in our analyses, as gender differences in math anxiety, generalized anxiety, and performance on the CRT are widely reported (e.g., Devine et al., 2012; Primi et al., 2018). Taken together, in the two experiments, we examined how math anxiety related to cognitive and metacognitive measures, beyond the effects of generalized anxiety, subjective numeracy, and gender, while manipulating both numerical content and mathematical reasoning demands.

## 2. Experiment 1

In Experiment 1, we examined factors influencing individuals’ success rates^1^, confidence, and overconfidence, when solving the numerical SAST puzzles and the CRT. In this experiment, in addition to the instructions informing about having unsolvable SAST matrices, we manipulated the allotted time frame. This time frame manipulation aimed at examining whether math anxiety effects are amplified under conditions of increased cognitive demand. Time constraints are known to increase cognitive load (Barrouillet et al., 2007; Paas & Van Merriënboer, 1994), and consequently impact math performance (Chan & Tang, 2020), as well as metacognitive monitoring and control (Sidi et al., 2017; Thiede & Dunlosky, 1999). Thus, employing time pressure may provide a context in which individual differences related to anxiety are more likely to manifest (Ashcraft & Krause, 2007).

### 2.1. Method

#### 2.1.1. Participants

To determine the sample size, we used a power calculation (GPower 3.1.9.7) for correlational analyses. Aiming for a medium effect size (*q* = 0.35), *α* = 0.05, and power = 0.80, the desired sample size was 208. Two hundred and twenty-three paid participants were recruited online through the Prolific platform (www.prolific.co). We invited native English speakers with approval rates above 90%. Exclusion criteria were based on items and participants. Valid trials were defined as not extraordinarily long (>240 s) and with a focus on the task window (rather than switching to another tab or window on the computer) for more than 50% of the measured response time. This led us to exclude 42 responses. We excluded participants who had fewer than 15 valid items (*N* = 3) or failed an attention check (*N* = 11). The analyzed data set included 209 participants (*Mage* = 27.6, *SD* = 8.4, 45.5% female), who completed 3712 trials.

#### 2.1.2. Materials

##### Numerical SAST

Participants received the SAST puzzles in a random order. After solving each puzzle, participants rated their confidence in their solution on a scale ranging from a “wild guess” to “definitely correct” (11–100 on a numerical scale). The internal consistency for the puzzle set was *Cronbach’s α* = 0.85, indicating high reliability.

##### Cognitive Reflection Test (CRT)

We utilized six open-ended CRT problems (Appendix B) generated as a less-familiar variation of the original CRT (Frederick, 2005), based on prior research (Primi et al., 2016; Shenhav et al., 2012). Here too, participants rated their confidence after solving each puzzle. Given the open-ended response format, the confidence scale ranged from 0–100%.

##### Subjective Numeracy Scale

The subjective numeracy scale reflects perceived quantitative abilities and aims at differentiating between individuals with low and high levels of numeracy (Fagerlin et al., 2007). We used the cognitive abilities subscale, which measures people’s belief of how well and how quickly they can perform numerical tasks (e.g., “*How good are you at working with fractions?*”). This 4-item subscale has been found to show strong correlations with objective numeracy (Fagerlin et al., 2007). Participants responded on a six-point Likert scale ranging from “*not at all good*” to “*extremely good*”. Higher scores indicate higher perceived numerical competence. Average ratings were used as the outcome measure. Internal consistency was good (*α* = 0.88).

##### Abbreviated Math Anxiety Scale (AMAS)

To measure math anxiety, we used the AMAS, which comprises two factors: learning math anxiety, which includes five items (e.g., *How anxious did you feel when you were: “Thinking about an upcoming math test one day before”*), and math evaluation anxiety, which includes four items (e.g., “*Listening to a lecture in math class*”; Hopko et al., 2003), to which participants had to respond based on their experiences at school. Each item was rated on a 5-point scale ranging from “*low anxiety*” to “*high anxiety*”. Higher scores indicate higher anxiety levels, and average ratings were used as the outcome measures. Internal consistency in the present study was excellent (*α* = 0.91).

##### Generalized Anxiety Disorder Scale (GAD-7)

To measure general anxiety, we used the GAD-7, which is a widely used, brief self-report scale intended to identify probable cases of GAD (Spitzer et al., 2006). The scale consists of seven items (e.g., “Over the last 2 weeks, how often have you been bothered by the following problems: *Feeling nervous, anxious or on edge.*”), rated on a four-point Likert scale ranging from “*not at all*” to “*nearly every day*”. Higher scores indicate higher anxiety levels. Average ratings were used as the outcome measure. Internal consistency in the present study was excellent (*α* = 0.91).

#### 2.1.3. Design and Procedure

The experimental structure was a 2 (notification: with vs. without) × 2 (time frame: time pressure vs. no pressure) between-participants design. After providing informed consent, participants received an explanation of the task rules and an example. Next, they viewed a worked example and completed two training puzzles. Then, participants solved the entire puzzle set. The two time frame conditions were run sequentially, due to a technical consideration related to the online platform: participants could only be directed to a single active study with a specified expected duration, whereas the two time-pressure conditions differed in their maximum study duration. Time frame was manipulated via the instructions, with no enforcement. Participants in the *time pressure* condition were invited to complete the study within 20 min, while those in the *no pressure* condition were invited for 30 min. Within each condition, participants were randomly allocated to a notification condition. Participants in the *with-notification* condition were informed that the task included some unsolvable puzzles, while participants in the *without-notification* condition received the instruction to give up on very difficult items, without being informed about the unsolvable puzzles. In both cases, the required response was “0”. Participants completed the CRT after the SAST. For each SAST and CRT item, participants rated their confidence using the relevant scale, 11–100% and 0–100%, respectively. Subsequently, they filled out the self-report questionnaires in the following order: subjective numeracy scale, AMAS, and GAD-7. Age and gender were provided by Prolific.

#### 2.1.4. SAST Performance Measures

##### Success Rates

Average success rate in the SAST.

##### Confidence

Average confidence across all SAST puzzles.

##### Overconfidence

Overconfidence was calculated for each participant as the difference between average confidence and success rate across task items. For example, if a participant solved 12 out of 18 SAST items correctly, their success rate was 66.7%. If the same participant’s mean confidence across these items was 80%, their overconfidence score was 13.3 percentage points (80 − 66.7). Positive values indicate overconfidence, whereas negative values indicate underconfidence.

#### 2.1.5. CRT Performance Measures

##### Success Rates

Average success rate in the CRT task.

##### Confidence

Average confidence across all CRT items.

##### Overconfidence

Overconfidence was calculated for each participant as the difference between average confidence and success rate across task items, as in the SAST.

#### 2.1.6. Ethical Approval

The study was approved by the institutional ethics review committee of the last author (#2022-024).

### 2.2. Results

Table 1 presents demographic characteristics and performance measures across the four groups. There were no differences between the four conditions in participants’ background variables, including their age (*p* = .766), gender (*p* = .177), subjective numeracy (*p* = .539), math anxiety (*p* = .670), and generalized anxiety (*p* = .414). The notification condition had no effect on any of the SAST performance measures (*p* > .146). A manipulation check confirmed that participants responded in line with the time-frame manipulation: mean response times (in seconds) were shorter in the time-pressure condition (*M* = 50.6, *SD* = 20.9) compared to the no pressure condition (*M* = 62.7, *SD* = 27.9), *F*(1,205) = 12.666, *MSE* = 584.778, *η*^2^ = 0.058, *p* < .001. Time frame also had a main effect on success rates, with lower scores in the time pressure condition (*M* = 67.9, *SD* = 21.5) compared to the no pressure condition (*M* = 73.8, *SD* = 17.3), *F*(1,205) = 4.436, *MSE* = 388.759, *η*^2^ = 0.021, *p* = .036. However, time frame did not affect confidence (*p* = .056), and there was no significant interaction with notification condition (*p* > .093). There were also no main effects or interactions between the experimental manipulations (notification condition and time frame) in the case of math anxiety (*p* > .254).

In all experimental groups, participants were overconfident (average overconfidence was significantly greater than zero) in both the SAST (all *ps* < 0.001) and CRT (*ps* < 0.001). Correlations between performance measures on the SAST and the CRT, and self-report measures can be found in Table 2.

#### 2.2.1. Predicting Success Rates, Confidence, and Overconfidence in the Numerical SAST

The correlation of success rates with self-report measures indicated that higher subjective numeracy and lower math anxiety were associated with higher task performance. To determine which measures predicted success rates, we ran a regression analysis, including notification and time frame condition, subjective numeracy, math anxiety, generalized anxiety, and gender as predictors (Table 3). Multicollinearity diagnostics indicated no concerns (all tolerance values > 0.675; all VIF values > 1.006). Results showed that time frame (no time pressure), higher subjective numeracy, and lower math anxiety predicted higher success rates on the numerical SAST, accounting for 14.4% of the variance. Notification condition, generalized anxiety, and gender were not significant predictors.

As for success rates, for confidence too, the correlation analysis indicated that higher numeracy and lower math anxiety were associated with higher confidence in the task (Table 2). A similar regression analysis was conducted on confidence (Table 3). Multicollinearity diagnostics indicated no concerns (all tolerance values > 0.675; all VIF values > 1.006). Higher subjective numeracy and lower math anxiety predicted higher confidence. Gender (being female) also predicted higher confidence, yet it was marginally significant (*p* = .05). Together, the variables accounted for 13% of the variance. Notification condition, time frame, and generalized anxiety were not significant predictors of confidence.

Finally, the same analysis was conducted on overconfidence (Table 3). The correlation analysis revealed no associations with self-report measures. Multicollinearity diagnostics indicated no concerns (all tolerance values > 0.675; all VIF values > 1.006). Higher math anxiety and lower generalized anxiety predicted a larger overconfidence bias, accounting for 3.2% of the variance, with a marginally significant model. Time frame, notification condition, subjective numeracy, and gender were not significant predictors.

#### 2.2.2. Predicting Success Rates, Confidence, and Overconfidence in the CRT

Correlation analyses indicated that higher numeracy and lower math anxiety were associated with higher success rates in the CRT (Table 2). For confidence, there was a similar pattern, although general anxiety also had a negative relationship. Finally, lower numeracy and lower math anxiety were correlated with a larger calibration bias. We conducted three regression analyses, including subjective numeracy, math anxiety, generalized anxiety, and gender, as predictors for success rates, confidence, and overconfidence in the CRT task (Table 4).

For success rate, multicollinearity diagnostics indicated no concerns (all tolerance values > 0.68; all VIF values > 1.08). Results showed that higher subjective numeracy and lower math anxiety predicted success rates accounting for 19.7% of the variance. Generalized anxiety and gender were not significant predictors.

For confidence, multicollinearity diagnostics indicated no concerns (all tolerance values > 0.68; all VIF values > 1.08). Results showed that higher subjective numeracy, lower math anxiety, and gender (male) predicted confidence, accounting for 19.6% of the variance. Generalized anxiety was not a significant predictor.

Finally, for overconfidence, multicollinearity diagnostics indicated no concerns (all tolerance values > 0.68; all VIF values > 1.08). Math anxiety was the only significant predictor of overconfidence, with higher math anxiety predicting more overconfidence, and accounting for 4.3% of the variance.

### 2.3. Discussion

Experiment 1 investigated how individual differences in math anxiety, generalized anxiety and subjective numeracy, as well as task design elements of time pressure and knowledge about unsolvable items, predicted success rates, confidence, and overconfidence in the numerical SAST and the CRT. Our findings confirmed the predictive power of math anxiety to success rates, confidence, and overconfidence across both tasks, after taking into account gender, generalized anxiety and subjective numeracy. The convergence between the two tasks implies that math anxiety is systematically related to lower success rates on reasoning problems that involve numbers, irrespective of whether the task involves mathematical reasoning and computations, as in the CRT, or not, as in the SAST.

The experimental manipulations yielded limited effects. Time pressure reduced accuracy, consistent with previous research (Sidi et al., 2017; Thiede & Dunlosky, 1999). However, in line with prior math anxiety research (e.g., Kellogg et al., 1999), time pressure did not interact with math anxiety, suggesting that its effects operated broadly across anxiety levels. The notification manipulation regarding the presence of unsolvable puzzles had no effect on performance measures and did not interact with math anxiety. This finding suggests that simple instructional interventions may not be sufficient to attenuate the impact of math anxiety in numerical reasoning tasks.

Overall, these findings support the relevance of math anxiety to confidence and the calibration of metacognitive judgments, in line with relevant theorizing (Morsanyi et al., 2019; Scheibe et al., 2023). As the results were very similar across the numerical SAST and the CRT, they suggest that the mere presence of numerical content, rather than the requirement to perform computations or numerical operations, was enough to trigger the negative effects of mathematics anxiety. In Experiment 2, we investigated the boundary conditions of these effects by contrasting a non-numerical variation of the SAST with the CRT.

## 3. Experiment 2

Experiment 2 also utilized the SAST, after replacing numbers with letters, thereby eliminating numerical cues while maintaining identical reasoning demands (Figure 1). Although time pressure in Experiment 1 led to lower overall accuracy, this effect did not interact with math anxiety. Thus, we did not include this manipulation in Experiment 2, and participants were invited for 30 min (the lengthier condition). However, we did include the notification manipulation informing participants about the presence of unsolvable matrices. We also administered the CRT in the same way as in Experiment 1 to replicate the relations between math anxiety and performance measures across studies and to examine their association with the non-numerical task within participants, while controlling for the effect of other individual-differences measures.

### 3.1. Method

#### 3.1.1. Participants

The sample size was set to be similar to Experiment 1. Thus, 220 participants were recruited online through the Prolific platform (www.prolific.co). Participants were required to be native English speakers, with a high approval rate on Prolific (>90%), with some experience in the platform (between 50 and 200 previous study participations). Exclusion criteria were similar to Experiment 1, excluding participants who had less than 15 valid items or failed an attention check (*N* = 20), leaving 200 participants in the analyzed data (*Mage* = 26.4, *SD* = 7.3, 45.7% female). Trials with extraordinarily long response times (RT > 240 s; 21 trials) or with a low focus score (focus < 0.50; 85 items) were excluded. This resulted in 3858 trials that were included in the analyses.

#### 3.1.2. Materials

##### Letter SAST

This task was based on the same design as the numerical SAST in Experiment 1, keeping the task as similar as possible. The only difference was that the numbers were replaced by the corresponding letters. For example, “1” was replaced with “A”, “2” was replaced with “B”, etc. (See Appendix A). The internal consistency for the puzzle set was *Cronbach’s α* = 0.87, indicating high reliability.

##### Subjective Numeracy Scale

The same scale was used as in Experiment 1. The internal consistency in the present study was good (*α* = 0.88).

##### Abbreviated Math Anxiety Scale (AMAS)

The same scale was used as in Experiment 1. The internal consistency in the present study was excellent (*α* = 0.90).

##### Generalized Anxiety Disorder Scale (GAD-7)

The same scale was used as in Experiment 1. The internal consistency in the present study was excellent (*α* = 0.90).

##### Cognitive Reflection Test (CRT)

The same task was used as in Experiment 1.

##### Design and Procedure

The design was identical to Experiment 1, except that the time-frame manipulation was omitted.

#### 3.1.3. Performance Measures

Performance measures for the letter SAST and the CRT were identical to Experiment 1.

### 3.2. Results

There were no differences between the two experimental conditions in gender distribution (*p* = .938), subjective numeracy (*p* = .898), math anxiety (*p* = .590), or generalized anxiety (*p* = .482), see Table 5. There was a significant age difference, *t*(198) = 2.146, *p* = .033, *Cohen’s d* = 0.304, such that participants in the with-notification condition were slightly older than those in the without-notification condition. Thus, age was included in all analyses. ANOVA analyses examined the effect of the notification conditions with age as a covariate and found no effect on any of the SAST performance measures (*p* > .343).

As in Experiment 1, across groups and tasks, the calibration bias was in the direction of overconfidence (all *p* values < .001). Correlation analysis (Table 6) found similar associations between the two tasks as in Experiment 1, with SAST accuracy and confidence positively correlated with CRT accuracy and confidence.

#### 3.2.1. Predicting Success Rates, Confidence, and Overconfidence in the Letter SAST

The correlation of success rates with self-report measures indicated that lower math anxiety was associated with higher success rates (Table 6). Because the individual-difference measures were conceptually related, for controlling for potentially alternative explanations, the regression analysis for success rates included notification condition, subjective numeracy, math anxiety, generalized anxiety, gender, and age as predictors (Table 7). Multicollinearity diagnostics indicated no concerns (all tolerance values > 0.737; all VIF values > 1.026). Results showed that only age (younger) predicted higher success rates, accounting for 4.4% of the variance. All other variables were not significant predictors.

An identical analysis was conducted on confidence in the SAST (Table 7). The correlation analysis indicated that higher numeracy, lower math anxiety, and lower general anxiety, were all associated with higher confidence in the task (Table 6). The Multicollinearity diagnostics indicated no concerns (all tolerance values > 0.737; all VIF values > 1.026). Results showed that higher subjective numeracy, lower generalized anxiety, and age (younger, marginally) predicted higher confidence, accounting for 8.5% of the variance. Notification condition, math anxiety, and gender were not significant predictors.

Finally, the same analysis was conducted on overconfidence (Table 7). As in Experiment 1, the correlation analysis found associations with the individual level measures (Table 6). Multicollinearity diagnostics indicated no concerns (all tolerance values > 0.735; all VIF values > 1.031). In this case, none of the variables were found to significantly predict overconfidence.

#### 3.2.2. Predicting Success Rate, Confidence, and Overconfidence in the CRT

Correlation analyses indicated that higher subjective numeracy, lower math anxiety, and lower generalized anxiety were associated with higher success rates and higher confidence in the CRT, while lower math anxiety was correlated with a larger calibration bias (Table 6). We conducted three regression analyses, including subjective numeracy, math anxiety, generalized anxiety, age, and gender, as predictors for success rates, confidence, and overconfidence in the CRT task (Table 8).

For success rates, multicollinearity diagnostics indicated no concerns (all tolerance values > 0.737; all VIF values > 1.019). Unlike with the letter SAST, higher subjective numeracy, lower math anxiety, and gender (male) predicted higher accuracy, accounting for 23% of the variance. Generalized anxiety and age were not significant predictors.

For confidence, multicollinearity diagnostics indicated no concerns (all tolerance values > 0.737; all VIF values > 1.019). Again, higher subjective numeracy, lower math anxiety, and gender (male) predicted confidence, accounting for 29.9% of the variance. Generalized anxiety and age were not significant predictors.

Finally, for overconfidence, multicollinearity diagnostics indicated no concerns (all tolerance values > 0.737; all VIF values > 1.019). Only math anxiety was a significant predictor of overconfidence, with higher math anxiety related to more overconfidence, accounting for 3% of the variance.

### 3.3. Discussion

Experiment 2 examined whether the associations between math anxiety, success rates, and metacognitive confidence and overconfidence observed in the numerical SAST would persist when numerical symbols were replaced by letters, while preserving the task’s logical structure and demands. The results suggest that replacing the numerical symbols with letters changed the pattern of predictors. In the letter SAST, math anxiety was correlated with lower success rates and confidence, but it did not uniquely predict success rates, confidence, or overconfidence once the other variables were included in the models. Success rates were predicted only by age, whereas confidence was predicted by higher subjective numeracy, lower generalized anxiety, and, marginally, by younger age. None of the predictors significantly explained overconfidence. Together, these findings suggest that the associations between math anxiety and metacognitive judgments found in the numerical SAST do not extend to a structurally similar task without numerical content.

At the same time, the CRT results were consistent with Experiment 1. In particular, math anxiety predicted lower success rates, lower confidence, and greater overconfidence. This replication supports the robustness of the association between math anxiety and both performance and metacognitive judgment in explicitly numerical reasoning tasks.

Finally, the notification manipulation, again, had no effect on success rates, confidence, or overconfidence. Thus, Experiment 2 strengthens the notion that informing participants about the possible presence of unsolvable items does not meaningfully alter performance measures.

## 4. General Discussion

The present study provides an empirical investigation of the relations between math anxiety and cognitive and metacognitive processes (see Morsanyi et al., 2019 and Scheibe et al., 2023 for theoretical discussions). Using a combination of the SAST and CRT tasks, we also examined within participants whether math anxiety is associated with performance measures and metacognitive monitoring in explicitly mathematical tasks only (i.e., the CRT), or whether it was also related to these indicators in reasoning tasks that contained numerical cues, but did not require computations (i.e., the numerical SAST), and tasks that did not involve numerical cues, but required the same reasoning processes (i.e., the letter SAST).

Across two experiments, we found that math anxiety was consistently associated with lower success rates, lower confidence, and higher overconfidence in tasks that included numerical content. This pattern emerged not only in the CRT, which required mathematical reasoning, but also in the numerical SAST, which did not require any computations or numerical reasoning. When the same Sudoku task structure was presented with letters instead of numbers, math anxiety no longer uniquely predicted success rates, confidence, or overconfidence. These findings suggest that the effects of math anxiety are not limited to computational tasks. Nevertheless, the results also do not reflect a general difficulty with reasoning among math anxious participants. Instead, the results suggest that the mere presence of numerical cues is enough to frame the task as math-related, and, thus, threatening for some people.

Beyond the above contributions, our study presents a new paradigm to investigate reasoning processes and metacognitive judgments in different domains in the form of the matched numerical and letter SAST. This approach offers flexibility in manipulating both the number of missing cells required for solution (which is closely related to objective difficulty), and the overall number of missing cells (which is related to subjective assessments of difficulty, especially at the start of the solution process), providing a useful tool to investigate the impact of perceived difficulty on solution strategies and metacognitive decisions. As they require a single solution, our tasks are also easy to score.

### 4.1. Math Anxiety and Metacognitive Processes

Research on mathematics anxiety has uncovered some of the mechanisms linking mathematics anxiety to lower mathematics performance. These include processes operating at the time of performing mathematical tasks (for example, a tendency for local avoidance by preferring shortcuts and quick solutions—e.g., Ashcraft & Faust, 1994; Beilock & DeCaro, 2007; Morsanyi et al., 2014; and a temporary reduction in available working memory resources in highly anxious individuals—e.g., Eysenck & Calvo, 1992; Luttenberger et al., 2018), as well as processes unfolding over a longer term (for example, developing higher anxiety as a result of negative experiences with math—e.g., Dowker et al., 2016; and reduced performance due to avoidance of non-compulsory mathematics practice and courses—e.g., Lau et al., 2024).

The current study extends previous work by not only showing a relationship between math anxiety and lower confidence (e.g., Rolison et al., 2016), but also between math anxiety and larger overconfidence. This effect was robust across the two numerical tasks, and operated above and beyond the effects of subjective numeracy, generalized anxiety, and gender. Thus, although math anxious people, on average, were less confident in their performance, they were more overconfident than other participants. This pattern of some acknowledgement of lower performance relative to others, but not enough, is similar to the classic Dunning-Kruger effect (see Pennycook et al., 2017, for a review), although prior research suggested that for the math domain low performers might be more modest than for other domains (Erickson & Heit, 2015).

A further potential implication of these findings is that math anxiety may serve as a (misleading) metacognitive cue (Ackerman, 2019), and/or operate via physiological reactions to numerical stimuli (Suárez-Pellicioni et al., 2016), using moment-to-moment assessments of math anxiety. In the current study, we measured trait-level math anxiety, which may not precisely reflect the level of anxiety experienced by participants while solving the tasks. Thus, future studies delving into math anxiety and metacognitive processes may utilize measures of state math anxiety to better assess the experience at the time of making metacognitive decisions.

Additional exploratory analyses of metacognitive resolution in the SAST are reported in the Supplementary Materials. Resolution values were positive, and moderate in strength, indicating that participants’ confidence judgments reliably discriminated between correct and incorrect responses in the case of both versions of the SAST. However, our regression analyses for the Numerical SAST indicated that none of the experimental manipulations, demographic or individual differences measured explained significant variance in metacognitive resolution in this task. In the case of the Letter SAST, only gender emerged as a significant predictor, with females showing lower resolution than males. Given the absence of established benchmarks and the limited understanding of individual-difference predictors of resolution, these findings are reported descriptively and are not a focus of the present study.

### 4.2. Scope and Boundaries for Math Anxiety

A central contribution of the present study is the finding that math anxiety relates to success rates and metacognitive monitoring when numerical stimuli are present, even when no explicit computations or mathematical reasoning are required. These findings are consistent with Rubinsten et al. (2015), who demonstrated attentional biases toward math-related stimuli among individuals with higher math anxiety, and extend this pattern to problem-solving performance and metacognitive monitoring. Notably, these effects did not extend to a structurally equivalent letter SAST, potentially indicating that the presence of numbers has a key role in triggering math anxiety. These results complement other evidence that shows that the association between math anxiety and math performance varies across numerical tasks (e.g., O’Connor et al., 2023), and it is also sensitive to within-task manipulations of task content (e.g., Mielicki et al., 2023). The present findings extend this work by isolating numerical symbols from computational requirements: the numerical SAST preserved numerical content without requiring arithmetic operations, whereas the letter SAST preserved the reasoning structure while removing numerical content. Thus, the results suggest that numerical content may be one task feature through which math anxiety becomes relevant to both performance and metacognitive monitoring.

A further notable finding is that whereas in the case of numerical tasks math anxiety predicted confidence beyond the effect of general anxiety and subjective numeracy (with general anxiety not making significant contributions), in the case of the letter SAST, general anxiety was a predictor of confidence, whilst math anxiety was not. This distinction demonstrates both the important links between anxiety and confidence in reasoning tasks, and the domain-specific effect of math anxiety.

### 4.3. Subjective Numeracy

The results also show the role of subjective numeracy, which reflects individuals’ self-perceived numerical competence (Fagerlin et al., 2007; Peters, 2012). In this study, subjective numeracy predicted success rates in the numerical tasks but not in the letter SAST. This pattern is consistent with the idea that subjective numeracy captures self-perceived numerical competence and is therefore most relevant when the task includes numerical content (Fagerlin et al., 2007; Liberali et al., 2012). However, subjective numeracy predicted confidence in the numerical SAST and CRT, and also in the letter SAST. This finding suggests that subjective numeracy, similar to math anxiety (Pearl et al., 2026; Scheibe et al., 2022), may operate as a domain-related metacognitive cue. Even when numerical symbols were removed, the letter SAST may still have resembled a task associated with numerical or formal reasoning. Participants with higher subjective numeracy may therefore have approached the task with a stronger sense of competence, which was reflected in their confidence judgments. Importantly, however, subjective numeracy did not predict overconfidence. Thus, it appears to be related to confidence itself rather than to monitoring accuracy.

### 4.4. The Role of Instructional Information

Across both experiments, the notification manipulation did not affect success rates, confidence, or overconfidence. Informing participants that some items might be unsolvable was predicted to inform the monitoring process and aid in calibrating participants’ judgments to their actual performance. Instead, overconfidence remained robust. This suggests that calibration errors in these reasoning tasks may be driven less by a priori task knowledge and more by the cues participants use while solving the problems, including math anxiety. In the present study, difficulty of the SAST was related to the number of empty cells within the puzzles, and, in particular, within the lines/columns of the target cells. An analysis of difficulty was beyond the scope of the present study, but future studies may utilize the Sudoku variations offered here to delve further into the distinction between difficulty and solvability and their associations with individual characteristics, such as math anxiety.

### 4.5. Gender Differences in Confidence and Metacognitive Accuracy

Gender differences are pervasive in math anxiety research (e.g., Dowker et al., 2016), with women, in general, reporting higher levels of anxiety than men. Furthermore, success rates on the CRT tend to be lower in women than in men (e.g., Frederick, 2005), although these differences have been found to be mediated by mathematical reasoning skills and math anxiety (Primi et al., 2018).

Women are also likely to display lower confidence in their mathematics skills (e.g., Ganley & Lubienski, 2016), although gender differences in confidence outside of mathematical reasoning are highly dependent on the context and domain investigated (e.g., Lundeberg et al., 1994). Among university students, McMurran et al. (2023) found that men were more confident in general in their math problem-solving ability than women, although the gender gap decreased with increasing math problem difficulty. Interestingly, the same study reported that women’s confidence judgments were better calibrated (i.e., they were less overconfident) than men, a finding that is directly relevant to our studies.

In the current study, we found a gender difference in confidence ratings on the CRT, with women displaying lower confidence than men in both studies after the effects of the other variables were taken into account. Additionally, in Study 2 only, we also found lower performance on the CRT among female participants, replicating previous findings (e.g., Primi et al., 2018). Nevertheless, in the case of the numerical SAST, there was a trend for higher confidence in women, suggesting that gender differences in confidence are task-dependent, in line with previous studies (e.g., Lundeberg et al., 1994). Importantly, we did not find evidence of gender differences in metacognitive monitoring once the effects of the other variables were taken into account.

### 4.6. Implications

Taken together, the findings suggest that math anxiety should be understood not only as a factor that interferes with mathematical performance, but also as a factor that shapes metacognitive monitoring in numerical contexts. Notably, the results show that math anxiety relates to reasoning even when the task does not require calculation, provided that numerical symbols are present. This pattern may support a numerical-framing explanation, indicating that numerical symbols can function as cues that activate math-anxiety-related processes. Namely, the effects of math anxiety extend beyond formal mathematics, but remain tied to the symbolic and contextual features that make a task “feel” mathematical.

More broadly, the findings contribute to research on metacognition by showing that confidence and calibration are guided not only by task performance, but also by domain-specific affective and self-evaluative cues. In the present context, the findings extend cue-utilization accounts of metacognitive monitoring (Koriat, 1997) by suggesting that affective responses tied to self-perceptions regarding the domain as a whole can shape monitoring judgments (Ackerman, 2019). Importantly, even expecting lower performance in line with self-perceptions does not ensure sufficient moderation of performance expectations, which may lead to miscalibration (here, overconfidence). However, the relationship between math anxiety and overconfidence was modest. This suggests that calibration bias cannot be explained by math anxiety alone, and/or that this relationship may not be linear. These possibilities deserve dedicated research.

Finally, the present study offers a novel methodology not only for metacognitive research, but also for cognitive and educational research. The SAST is a highly flexible, customizable paradigm that bridges the gap between ecologically valid problem-solving and rigorous experimental control. By isolating a single target cell within a partially completed structure, the task allows researchers to manipulate specific cognitive load (e.g., time pressure), heuristic cues (e.g., empty cells’ visual arrangement), and difficulty levels (e.g., matrix size and number of missing cells). Future research could also use the SAST to examine whether the association between mathematics anxiety and metacognitive calibration varies across difficulty levels (e.g., by having more/less missing cells), while distinguishing difficult but solvable items and unsolvable items. Crucially, because the task strips away the tedious, time-consuming execution phase of traditional puzzles in favor of a single-keystroke response, it provides purer and more standardized data. This opens up new avenues for cognitive and educational researchers to delve into information-search strategies. Furthermore, the seamless structural equivalence between the numerical and letter-based variations provides a clean template for researchers wishing to isolate domain-specific anxieties or cognitive biases from general fluid reasoning abilities. Ultimately, the SAST offers a standardized, easily automatable tool for investigating the interplay between objective performance, subjective experiences (e.g., mental effort, Hoch et al., 2023), and effort regulation in experimental settings.

### 4.7. Additional Future Directions

Although the numerical and letter SAST were structurally similar, replacing numbers with letters may have changed more than numerical content alone. Indeed, although letters of the alphabet are likely to be as familiar as one-digit numbers, and people may be familiar with the relative ordinal position of letters, they would not immediately know which letters are included in the first 9 letters of the alphabet (for example, is “I” included?), making it harder to identify the missing letters in the SAST puzzles. Indeed, success rates in the letter SAST were considerably lower than in the numerical SAST. The numerical and letter SAST tasks were also administered in two separate studies. This was done to keep the study administration time manageable. Making direct comparisons between performance on the two types of tasks is a desirable next step. Future studies could examine the effects of symbolic formats in additional tasks to isolate more precisely which features activate math-anxiety-related processes.

Second, both experiments were conducted online with adult Prolific participants and relied on relatively brief reasoning tasks. Therefore, conclusions regarding educational or classroom contexts should be made cautiously. It remains unclear whether similar patterns would emerge among school or university students, or in learning contexts, classroom problem-solving, and high-stakes assessment situations. In such settings, math anxiety may be shaped by social and emotional experiences, such as repeated failure, negative feedback, evaluative pressure, and emphasis on speed and accuracy, and may also relate to persistence, avoidance, strategy use, and help-seeking in ways that are not fully captured by short experimental tasks (Dowker et al., 2016; Luttenberger et al., 2018; Ramirez et al., 2018). Future studies should therefore examine the present findings in educational samples and in more ecologically embedded learning contexts.

Third, in the present study, math anxiety and generalized anxiety were measured using self-report questionnaires, focusing on trait-like individual differences. These measures are appropriate for assessing more stable tendencies, but they do not capture momentary changes in anxiety during task performance. Distinguishing between trait and state anxiety is important, as these measures show differential relationships with other constructs (e.g., Orbach et al., 2019). Future research could combine self-report measures with physiological or behavioral indicators of anxiety (see Suárez-Pellicioni et al., 2016 for a review), such as pupil dilation, heart-rate variability, skin conductance, or facial expressions, to examine whether numerical cues elicit online affective responses while participants solve the task.

Fourth, although the findings show that math anxiety is associated with performance, confidence, and overconfidence in numerical contexts, they do not establish the causal direction of these associations. Math anxiety may affect monitoring and performance, but repeated experiences of miscalibration may also contribute to the development and maintenance of math anxiety. Longitudinal and experimental studies are needed to better understand causal relations, and how these relations unfold over time.

Fifth, we focused on the relations between math anxiety and final confidence, without providing insight into the ongoing metacognitive monitoring and control processes while task performance unfolded. This may be done in future studies by asking for initial judgments of solvability and/or multiple confidence judgments during the solution process or giving the option to skip items, for example (see Ackerman, 2014; Lauterman & Ackerman, 2024; Morsanyi et al., 2019). This research direction is also supported by work on emotion and metacognition in arithmetic, showing that negative affect can influence metacognitive judgments and strategy-related regulation, but that these effects depend on how and when metacognition is assessed (Geurten & Lemaire, 2022, 2024). For example, affective influences may be more evident in indirect or time-constrained judgments than in final, more deliberative confidence ratings. Other studies have included process indicators, like eye tracking and changes in pupil dilation (Dhengre et al., 2026; Purcell et al., 2023). Ongoing process tracking could shed light on the timeline of the effects of math anxiety on confidence, and how this influence contributes to calibration bias.

Sixth, the present study focused on metacognitive monitoring, operationalized through confidence judgments and calibration bias. Although monitoring is a prerequisite for effective self-regulation, it does not capture how individuals subsequently adapt their behavior in response to their metacognitive evaluations. Future research should therefore examine metacognitive control processes, such as strategy selection, effort allocation, and response revision, to provide a more comprehensive understanding of how mathematics anxiety influences metacognitive regulation.

Future studies could also examine response time as an additional process measure. In the present study, response time was used primarily to verify the time-pressure manipulation and identify invalid trials, rather than as an outcome measure. Because longer response times may reflect greater effort, higher task difficulty, inefficient processing, or distraction, the current design does not allow these possibilities to be disentangled. Future work combining response time with other process measures beyond confidence judgments may provide further insight into how mathematics anxiety influences metacognitive regulation during problem solving.

Finally, the current findings may be useful in planning future intervention-oriented research. For example, educational studies could examine whether reducing unnecessary numerical framing or offering feedback about confidence accuracy improves monitoring and regulation among learners with different levels of math anxiety. Applying the present metacognitive approach in school and university settings may help open current black boxes in studies that identify associations between math anxiety and achievement but do not reveal the underlying processes that lead to these outcomes. Shedding light on these processes can guide intervention design and define the conditions that allow individuals to overcome the pitfalls they are prone to.

### 4.8. Conclusions

Across two experiments, math anxiety was associated with lower performance, lower confidence, and greater overconfidence in tasks including numerical content. Importantly, these effects emerged even when no calculations were required, but were absent in a structurally equivalent letter-based task. These findings suggest that the influence of math anxiety extends beyond computation and may be triggered by numerical cues that frame a task as mathematical. More broadly, the results highlight the role of domain-specific affective factors in shaping both reasoning performance and metacognitive monitoring. Specifically, the results suggest that math anxiety functions as a metacognitive cue that shapes confidence judgments, highlighting the importance of considering affective factors when examining meta-reasoning processes (Ackerman & Thompson, 2017; Morsanyi et al., 2019).

From an educational perspective, the findings suggest that learners’ difficulties in mathematics-related contexts may arise not only from computational demands, but also from the way tasks are framed and perceived. This has implications for intervention design. Attempts to reduce the impact of mathematics anxiety may benefit from addressing students’ metacognitive monitoring directly, for example by helping them distinguish between affective reactions to numerical cues and actual task difficulty. Interventions may also consider whether numerical framing is necessary for a given learning goal, and how confidence, calibration, and strategy use can be supported when numerical content is unavoidable.

## Figures and Tables

**Figure 1:**
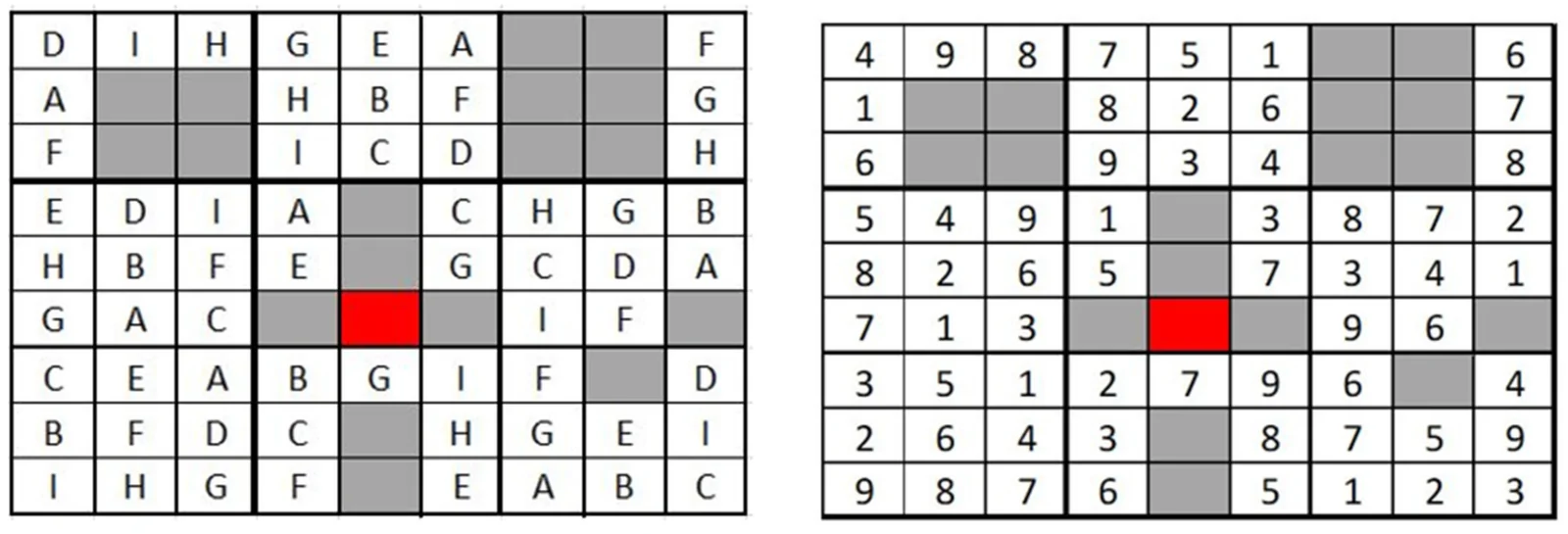
Examples of matched, numerical and letter Single-Answer Sudoku Task (SAST) items developed for the present study. Grey cells indicate additional blank, non-target cells, and the red cell indicates the target cell to be completed.

**Table 1:** Descriptive statistics for demographic and self-report variables across experimental groups.

	Pressured with Notification	Pressured Without Notification	No Pressure with Notification	No Pressure Without Notification
	*N* = 55	*N* = 61	*N* = 48	*N* = 45
Gender	38% female	39% female	56% female	51% female
	M (SD)	M (SD)	M (SD)	M (SD)
Age	26.6 (7.7)	28.1 (4.4)	27.5 (4.3)	28.1 (4.1)
Subjective numeracy	4.2 (1.2)	4.4 (1.1)	4.3 (1)	4.1 (1.3)
Math anxiety	2.9 (0.9)	2.7 (0.9)	2.7 (0.9)	2.8 (0.9)
Generalized anxiety	1.4 (0.7)	1.2 (0.8)	1.1 (0.8)	1.2 (0.7)
SAST success rates	66.9 (23.8)	68.9 (19.3)	77 (14.8)	70.5 (19.2)
SAST confidence	79.7 (17.3)	81 (16.4)	87.4 (10.3)	81.5 (15.4)
SAST overconfidence	12.7 (16.1)	12.1 (15)	10.4 (13.1)	11 (16.5)
CRT success rates	52.7 (28.8)	57.1 (30.8)	51.7 (29.8)	55.9 (29.9)
CRT confidence	79.1 (15.9)	77.5 (18.6)	76.1 (20)	77.5 (18.3)
CRT overconfidence	26.4 (27.3)	20.4 (27.5)	24.3 (22.8)	21.5 (27.3)

**Table 2:** Participant-level Pearson correlations between performance measures on the Numerical SAST and CRT, and the self-report scales.

			1	2	3	4	5	6	7	8
SAST	1	Success rates	--							
	2	Confidence	0.66 **	--						
	3	Overconfidence	−0.64 **	0.15 *	--					
CRT	4	Success rates	0.34 **	0.28 **	−0.17 *	--				
	5	Confidence	0.08	0.26 **	0.16 *	0.48 **	--			
	6	Overconfidence	−0.34 **	−0.14 *	0.30 **	−0.80 **	0.14 *	--		
Self-report scales	7	Subjective Numeracy	0.29 **	0.29 **	−0.09	0.36 **	0.33 **	−0.18 **	--	
	8	Math anxiety	−0.33 **	−0.29 **	0.13	−0.42 **	−0.37 **	−0.22 **	−0.51 **	--
	9	Generalized anxiety	0.01	−0.10	−0.11	−0.11	−0.17 *	−0.01	−0.13	0.31 **

*Note.* Boldface indicates statistically significant correlations. * <0.05, ** <0.001.

**Table 3:** Linear Regressions Predicting Success Rates, Confidence, and Overconfidence in the Numerical SAST.

Dependent Variable	Predictor	*B*	*t*	*p*	*F*	*Adj_R* ^2^	*df*	*p*
Success rates	Notification	−0.05	−0.8	.422				
	Time frame	0.16	2.43	**.016**				
	Subjective Numeracy	0.18	2.36	**.019**				
	Math anxiety	−0.28	−3.53	**<.001**				
	Generalized anxiety	0.12	1.78	.076				
	Gender	0.02	0.25	.801				
	Model				6.83	0.14	6202	<.001
Confidence	Notification	−0.07	−1.14	.255				
	Time frame	0.12	1.82	.071				
	Subjective Numeracy	0.23	2.96	**.003**				
	Math anxiety	−0.19	−2.41	**.017**				
	Generalized anxiety	−0.04	−0.50	.615				
	Gender	0.14	1.98	.050				
	Model				6.22	0.13	6202	<.001
Overconfidence	Notification	−0.01	−0.11	.917				
	Time frame	−0.01	−0.26	.210				
	Subjective Numeracy	−0.01	−0.06	.949				
	Math anxiety	0.17	2.04	**.043**				
	Generalized anxiety	−0.12	−2.69	**.008**				
	Gender	0.11	1.59	.113				
	Model				2.13	0.03	6202	.051

*Note.* Boldface indicates statistically significant predictors.

**Table 4:** Linear Regressions Predicting Success Rates, Confidence, and Overconfidence in the CRT.

Dependent Variable	Predictor	*β*	*t*	*p*	*F*	*Adj_R* ^2^	*df*	*p*
Success rates	Subjective numeracy	0.19	2.53	**.012**				
	Math anxiety	−0.32	−4.32	**<.001**				
	Generalized anxiety	0.02	0.36	.719				
	Gender	−0.06	−0.93	.354				
	Model				13.72	0.19	4204	<.001
Confidence	Subjective numeracy	0.16	2.17	**.031**				
	Math anxiety	−0.25	−3.3	**.001**				
	Generalized anxiety	−0.03	−0.48	.634				
	Gender	−0.17	−2.67	**.008**				
	Model				12.45	0.19	4204	<.001
Overconfidence	Subjective numeracy	−0.01	−1.24	.218				
	Math anxiety	0.19	2.37	**.019**				
	Generalized anxiety	−0.05	−0.68	.499				
	Gender	−0.05	−0.74	.461				
	Model				3.36	0.043	4204	.011

*Note.* Boldface indicates statistically significant predictors.

**Table 5:** Demographic variables and dependent measures across experimental groups.

	With Notification	Without Notification
	*N* = 101	*N* = 99
Gender	46% female	45.5% female
	M (SD)	M (SD)
Age	27.4 (7.8)	25.3 (6.5)
Subjective Numeracy	4.2 (1.1)	4.2 (1.2)
Math anxiety	2.8 (0.8)	2.7 (0.9)
Generalized anxiety	1.3 (0.8)	1.3 (0.7)
SAST success rates	57.5 (26.3)	60.7 (23.6)
SAST confidence	76.4 (17.6)	77.7 (15.4)
SAST overconfidence	18.9 (21.4)	17 (17.6)
CRT success rates	54 (28.9)	56.2 (28.4)
CRT confidence	76.7 (18.6)	76.8 (17.9)
CRT overconfidence	22.8 (26.2)	20.5 (27.4)

**Table 6:** Participant-level Pearson correlations between performance measures on the Letter SAST, CRT, and self-report scales.

			1	2	3	4	5	6	7	8
Performance measures SAST	1	Success rates								
	2	Confidence	0.62 **							
	3	Overconfidence	−0.75 **	0.05						
Performance measures CRT	4	Success rates	0.41 **	0.36 **	−0.22 **					
	5	Confidence	0.01	0.29 **	0.23 **	0.42 **				
	6	Overconfidence	−0.43 **	−0.19 **	0.39 **	−0.79 **	0.24 **			
Self-report scales	7	Subjective Numeracy	0.09	0.21 **	0.07	0.35 **	0.50 **	−0.03		
	8	Math anxiety	−0.17 *	−0.19 **	0.05	−0.44 **	−0.37 **	0.22 *	−0.42 **	
	9	Generalized anxiety	−0.12	−0.21 **	−0.03	−0.17 *	−0.14 *	0.09	−0.08	0.32 **

*Note.* Boldface indicates statistically significant correlations. * < 0.05, ** < 0.001.

**Table 7:** Linear Regressions Predicting Success rates, Confidence, and Overconfidence in the Letter SAST.

Dependent Variable	Predictor	*β*	*t*	*p*	*F*	*Adj_R* ^2^	*df*	*p*
Success rates	Notification	0.04	0.56	.574				
	Subjective numeracy	0.05	0.67	.504				
	Math anxiety	−0.13	−1.65	.101				
	Generalized anxiety	−0.1	−1.32	.187				
	Gender	0.13	1.84	.068				
	Age	−0.15	−2.04	**.043**				
	Model				2.52	0.04	6192	.023
Confidence	Notification	0.02	0.21	.833				
	Subjective numeracy	0.19	2.41	**.017**				
	Math anxiety	−0.06	−0.74	.460				
	Generalized anxiety	−0.21	−2.85	**.005**				
	Gender	0.06	0.78	.434				
	Age	−0.14	−1.97	.050				
	Model				4.051	0.085	6192	<.001
Overconfidence	Notification	−0.04	−0.53	.594				
	Subjective Numeracy	0.09	1.11	.268				
	Math anxiety	0.12	1.46	.145				
	Generalized anxiety	−0.05	−0.65	.518				
	Gender	−0.12	−1.66	.098				
	Age	0.07	0.69	.340				
	Model				1.19	0.006	6192	.314

*Note.* Boldface indicates statistically significant predictors.

**Table 8:** Linear Regressions Predicting Success rates, Confidence, and Overconfidence in the CRT.

Dependent Variable	Predictor	*β*	*t*	*p*	*F*	*Adj_R* ^2^	*df*	*p*
Success rates	Subjective numeracy	0.16	2.33	**.021**				
	Math anxiety	−0.34	−4.66	**<.001**				
	Generalized anxiety	−0.04	−0.62	.531				
	Gender	−0.15	−2.38	**.018**				
	Age	0.03	0.56	.578				
	Model				12.79	0.230	5193	<.001
Confidence	Subjective numeracy	0.37	5.58	**<.001**				
	Math anxiety	−0.17	−2.42	**.016**				
	Generalized anxiety	−0.05	−0.78	.435				
	Gender	−0.20	−3.27	**.001**				
	Age	0.03	0.58	.562				
	Model				17.89	0.299	5193	<.001
Overconfidence	Subjective numeracy	0.08	1.01	.314				
	Math anxiety	0.25	3.03	**.003**				
	Generalized anxiety	0.01	0.14	.885				
	Gender	0.03	0.37	.708				
	Age	−0.01	−0.19	.846				
	Model				2.21	0.030	5193	.055

*Note.* Boldface indicates statistically significant predictors.

**Figure A1:**
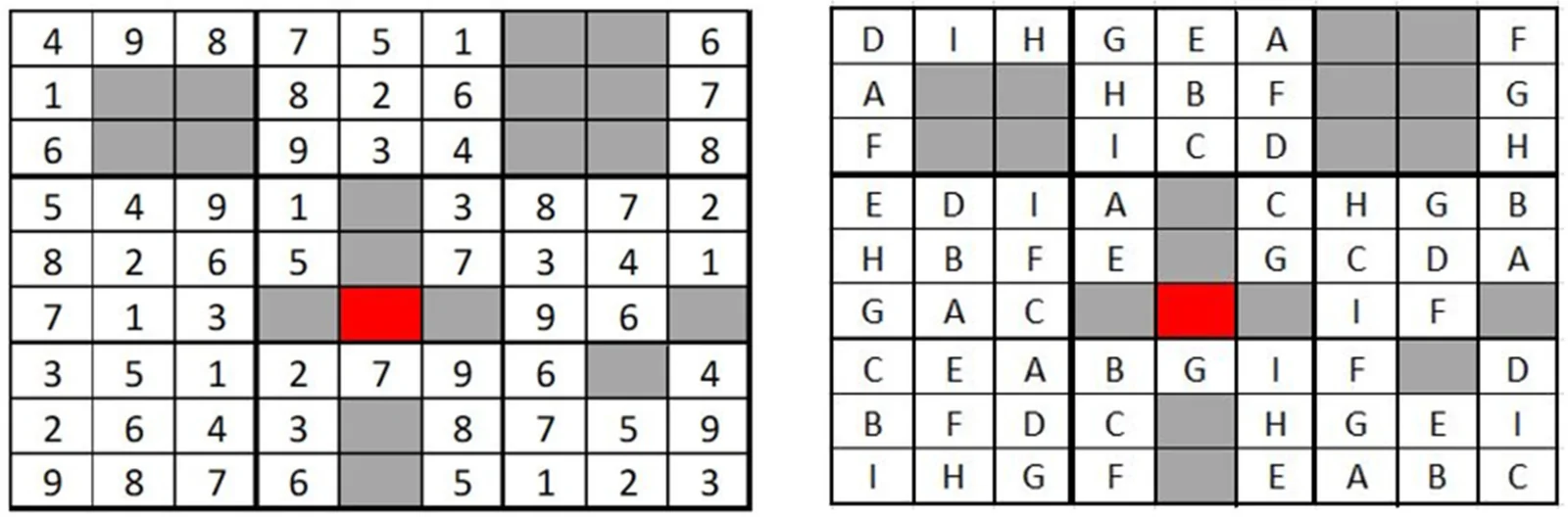
Puzzles from the numerical SAST (**left**) and the letter SAST (**right**). Grey cells indicate additional blank, non-target cells, and the red cell indicates the target cell to be completed.

## Data Availability

Data will be available upon request.

## Note

1 The term “success rate” refers here to the objective score on the task, that is, the degree to which participants’ responses match the correct solution. We use this term rather than “performance” or “accuracy”, because performance may also refer to process-based indicators, such as strategy use, efficiency, or physiological measures, and because accuracy is commonly used in metacognition research to refer to monitoring accuracy or calibration.

## Supplementary Materials

The following supporting information can be downloaded at: https://www.mdpi.com/article/10.3390/jintelligence14100237/s1. The full set of puzzles and their level of difficulty and solvability appears in: https://osf.io/2tvxw/ (accessed on 9 September 2026). Exploratory analyses of metacognitive resolution in the SAST are provided in the Supplementary Materials. Table S1: Linear Regressions Predicting Resolution in the Numerical SAST Task; Table S2: Linear Regressions Predicting Resolution in the Letter SAST Task.

## Abbreviations

The following abbreviations are used in this manuscript:

SAST	The Single-Answer Sudoku Task
CRT	Cognitive Reflection Test
AMAS	Abbreviated Math Anxiety Scale
GAD-7	Generalized Anxiety Disorder scale
RAMPS	Regulated Attention in Mathematical Problem-Solving
RT	Response time

## Appendix A. The Single-Answer Sudoku Task (SAST)

The classic 9 × 9 Sudoku puzzle is presented as an empty matrix, and respondents are required to fill in the entire matrix. The Single-Answer Sudoku Task (SAST) was developed to transform a lengthy, recreational puzzle into a highly controlled, standardized cognitive task suitable for cognitive and metacognitive experimentation. In contrast to traditional reasoning tasks, which often involve verbal problem representations, arithmetic procedures, or open-ended solution paths, the SAST preserves a constrained, rule-based reasoning structure, while allowing content to be manipulated to involve or eliminate numbers. The SAST matrix is presented with the majority of the cells already completed. Participants are required to fill in only a single, specific target cell marked in red. See Figure A1.

The primary design goals were twofold:

Scoring and responding efficiency: Instead of requiring full-grid completion or complex input procedures, participants respond by entering a single digit (or letter). This streamlined, brief responding procedure ensures that scoring is fully automated. More importantly, it minimizes the motor and procedural demands of the interface, allowing for a measurement of thinking times without contamination from complex input execution.

Eliciting uncertainty: Leaving some surrounding areas incomplete allows designing the task difficulty, by the amount and location of the empty cells. Moreover, for a metacognitive experiment, the matrix can be designed to require only some of the empty cells to be identified to solve the red cell. This design feature allows planning the uncertainty levels the answers are expected to be provided with. Furthermore, for pushing uncertainty even further, we included in the stimuli set 10% of the items which are non-solvable, as they result in the same digit more than once in the same line or not at all.

For calibrating item difficulty, success rates, and solvability constraints across the final puzzle set, we conducted two pre-test rounds online via Prolific. The first pre-test was intended to determine the number of SAST puzzles that could be completed in ~20 min and select the puzzles that fit the difficulty levels in the range of 20–80% success rates, and a few verification items (very high success rates). Eighty-nine participants completed the task, which included 22 puzzles in two conditions—with and without confidence ratings. Three participants were excluded for very low focus, and two were excluded for chance success rates and brief responding, leaving 84 participants in the analysis (*Mage* = 27.6, *SD* = 8.9, 49% female). A comparison of the difference between the conditions in response times (RTs) and focus on the application window revealed no differences (all *p* values > 0.148). However, confidence was relatively high (*M* = 89.1, *SD* = 13.4) and focus on the window was low (<0.5) for over 100 trials, which were mostly correct, indicating that participants might have taken notes to achieve a high confidence level in their solutions. Finally, time to finish the task was longer than expected, with an average of 32.3 min.

Thus, a second pre-test was conducted, with some changes. First, the instructions included a requirement to avoid taking notes. Second, a control question was added regarding solving aids used for solving the puzzles (e.g., computerized notes, pen and paper). Eighty-five participants completed the task. Five participants were excluded for very low focus or technical problems, leaving 80 participants in the analysis (*Mage* = 26.2, *SD* = 7.4, 38% female). Six trials with very long RT’s (>240 s) were excluded. A comparison of the difference between the conditions in accuracy, RT and focus revealed no differences (all *p* values > .436). Taken together with the first pre-test, this finding suggested that confidence ratings did not affect the relevant outcomes. However, the task still took longer than expected (=35.8 min). Moreover, more than 10 puzzles had higher than 80% success rates. Finally, confidence levels remained high (*M* = 82.8, *SD* = 17.1). Thus, for the final set, the task was shortened by excluding some of the easier puzzles, and three puzzles were replaced with unsolvable puzzles, to increase uncertainty and consequently the variance in confidence judgments. The stimuli used for the experiment included eighteen puzzles, comprising three verification puzzles randomly ordered in three set locations, three unsolvable items randomly ordered in three set locations, and twelve puzzles with success levels below 80% randomly ordered. The internal consistency for the entire set was good (*Cronbach’s α* = 0.848). For the solvable items, the number of grey cells correlated significantly with success rates (*r* = 0.769, *p* < .001), confidence (*r* = 0.825, *p* < .001), and RT (*r* = 0.879, *p* < .001), suggesting that puzzles with more grey cells were more demanding, eliciting longer solution times, lower success, and reduced confidence. These pre-test findings support the construct validity of the SAST by showing that item difficulty varied systematically with the intended structural manipulation, rather than reflecting arbitrary differences between puzzles. Construct validity was further supported by the moderate correlations between the numerical SAST and related constructs, such as the CRT, subjective numeracy and math anxiety, and weaker correlations with general anxiety (Table 2).

To create the letter task for Experiment 2, the numbers were replaced with their corresponding letters from the English alphabet (1 → A, 2 → B, 3 → C, 4 → D, 5 → E, 6 → F, 7 → G, 8 → H, 9 → I). This transformation preserved the rule structure, target-cell response format, and solvability status of the puzzles, while removing numerical symbols. The letter version therefore provided a direct comparison condition for examining whether the effects observed in the numerical SAST reflected numerical content or the broader logical structure of the task. See examples in Figure A1.

The full set of puzzles and their level of difficulty and solvability appears in: https://osf.io/2tvxw/ (accessed on 9 September 2026).

## Appendix B. CRT Items

(1) The ages of Mark and Adam add up to 28 years in total. Mark is 20 years older than Adam. How old is Adam?
  ____________ years

(2) If it takes 10 s for 10 printers to print out 10 pages of paper, how many seconds will it take 50 printers to print out 50 pages of paper?
  ____________ seconds

(3) On a loaf of bread, there is a patch of mould. Every day, the patch doubles in size. If it takes 22 days for the patch to cover the entire loaf of bread, how many days would it take for the patch to cover half of the loaf of bread? ___ days
(4) If three elves can wrap three toys in 1 h, how many elves are needed to wrap six toys in 2 h?
  _____________elves

(5) Jerry received both the 15th highest and the 15th lowest mark in the class. How many students are there in the class?
  ___________students

(6) In an athletics team, tall members are three times more likely to win a medal than short members. This year the team has won 60 medals so far. How many of these have been won by short athletes?
  ____________medals
