# Supplementary material for: Math Anxiety and Metacognition: How Numerical Content Shapes Confidence
Source: J Intell. 2026 Oct 2;14(10):237. doi: 10.3390/jintelligence14100237 (PMC13706537; doi:10.3390/jintelligence14100237)
Supplement: Supplementary file 1 [file jintelligence-14-00237-s001.zip › jintelligence-4444024-supplementary.pdf]

Supplementary Analysis: Metacognitive Resolution in the SAST

Metacognitive resolution reflects the extent to which participants discriminate between correct and incorrect responses in their confidence judgments. Resolution was calculated for each participant as the Goodman–Kruskal gamma correlation between item-level success and confidence. Resolution is a relative subjective accuracy measure, in which positive values indicate that confidence tended to be higher for correct than incorrect responses. Its magnitude has no established benchmark and is influenced by task characteristics. Accordingly, the analyses below are reported descriptively and should be interpreted cautiously.

Experiment 1.

Resolution scores were available for 190 participants. It was unavailable when there was insufficient within-participant variability in accuracy and/or confidence ratings to estimate a gamma correlation. Mean resolution was .50 (*SD* = .54) in the no-notification/time-pressure condition, .57 (*SD* = .58) in the no-notification/no-pressure condition, .43 (*SD* = .55) in the notification/time-pressure condition, and .64 (*SD* = .33) in the notification/no-pressure condition.

A regression analysis examined whether resolution was predicted by notification condition, time frame, subjective numeracy, math anxiety, generalized anxiety, and gender. None of the predictors significantly predicted resolution (all *p*s ≥ .068), see Table S1.

Table S1. Linear Regressions Predicting Resolution in the Numerical SAST Task.

Dependent variable	Predictor	*β*	*t*	*p*	*F*	*Adj_ R^2*	*df*	*p*
Resolution	Notification	.023	.32	.749				
	Time frame	.136	1.84	.068				
	Subjective Numeracy	-.098	-1.14	.258				
	Math anxiety	.063	.70	.483				
	Generalized anxiety	.002	0.03	.975				
	Gender	-.118	-1.52	.130				
	Model				1.34	.011	6,183	.243

Experiment 2.

Resolution scores were available for 187 participants. As in Experiment 1, it was unavailable when there was insufficient within-participant variability in accuracy and/or confidence ratings to estimate a gamma correlation. Mean resolution was .57 (*SD* = .38) in the no-notification condition and .50 (*SD* = .42) in the notification condition.

A regression analysis examined whether resolution was predicted by notification condition, subjective numeracy, math anxiety, generalized anxiety, gender, and age (Table S2). Gender was the only statistically significant predictor in Experiment 2. This isolated exploratory finding is not interpreted further given the modest variance explained by the model and the limited evidence concerning predictors of resolution. Replication is needed before further consideration.

Table S2. Linear Regressions Predicting Resolution in the Letter SAST Task.

Dependent variable	Predictor	*β*	*t*	*p*	*F*	*Adj_ R^2*	*df*	*p*
Resolution	Notification	.079	1.08	.280				
	Subjective Numeracy	-.085	-1.06	.291				
	Math anxiety	-.164	-1.97	.051				
	Generalized anxiety	.138	1.78	.076				
	Gender	-.163	-2.21	.029				
	Age	-.077	-1.04	.298				
	Model				2.36	.042	6,179	.032
